# Preliminary Assessment of p-Anisidinium-Based Ionic Solids as Potential DNA Staining Agents: DFT and Molecular Docking Studies

**DOI:** 10.1007/s10895-026-04872-8

**Published:** 2026-07-21

**Authors:** Vuyolwethu Tokoyi, Eric Oluwafisayo Akintemi, Hadley S. Clayton, Nirmala Deenadayalu

**Affiliations:** 1 Department of Chemistry, Faculty of Applied Sciences, Durban University of Technology, Durban, South Africa; 2 Department of Chemistry, University of South Africa, Florida Science Campus, Johannesburg, 1709 South Africa

**Keywords:** p-anisidinium, Ionic solids (ISs), UV-Vis absorption, DNA-staining, DFT

## Abstract

This work involved the preparation and characterization of a variety of p-anisidinium-based ionic solids (ISs) exhibiting significant DNA-staining capabilities, as guided and evidenced by their observed chromogenic behavior in ethanol under UV–Vis absorption. The obtained structural and functional group elucidation results verified the successful preparation of the ISs (^1^H and ^13^C-NMR, and FTIR), the significant role played by different anions on their overall colour compared to the pristine material was validated, and this was attributed to the aniline chromophore moiety and electronic interaction between the cation and anion. The UV-Vis absorption spectra illustrated three characteristic bands attributed to π→π*, n→π*, and intermolecular anion-cation charge-transfer-transition (CTT) commonly observed for p-anisidinium-based compound. Gel electrophoresis analysis was conducted to examine the potential of the ISs as novel alternatives that can be used either as loading or fluorescent DNA staining dyes. Among the examined, [p-Anis]^+^[NO_2_]^−^ was illustrated as a potent alternative loading and fluorescent DNA-staining dye. This observation was further validated with DFT simulation using b3lyp/6-311 + + g(d, p) method, and gave energy gap 3.40 eV, suggesting good chemical reactivity and moderate electronic conductivity, with global electrophilicity index value of 3.41 eV suggesting low stabilization energy and high susceptibility to charge transfer. The obtained ADME parameters suggest that this compound is soluble in water, have good drug likeness, supporting its DNS binding affinity, and a potential orally active drug since it obeys both Lipinski and Verber rules. The binding energies from the molecular docking simulations are − 6.9 and − 4.9 kcal/mol for ciprofloxacin and [p-Anis]^+^[NO_2_]^−^, respectively against *Escherichia coli*; and − 5.7 and − 5.9 kcal/mol for metronidazole and [p-Anis]^+^[NO_2_]^−^, respectively against *Clostridium difficile*. Key residues Met404 and Ala429 are common in both pairs for *E. coli* while Arg272 and Asp285 in both pairs *C. difficile*.

**Graphical Abstract:**

**Figure:**
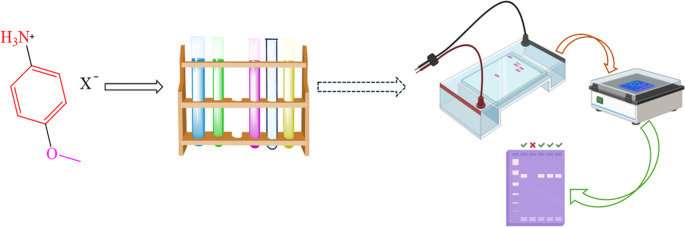
Pathway synthesis, optical behavior, and preliminary application of p-Anisidinium ionic solids for DNA staining

**Supplementary Information:**

The online version contains supplementary material available at 10.1007/s10895-026-04872-8.

## Introduction

Chemistry of ionic liquids (ILs) is dominated by electron and proton transfer, but the activity of protons that lead to solid and liquid state products is poorly understood [1]. In 1914, P. Walden reported the first successfully synthesized ionic liquid (IL) namely ethylammonium nitrate [2, 3]. He observed that this unusual combination of an organic cation and an inorganic anion, resulted in an asymmetric salt with a melting point of ~ 12 °C, substantially lower than the traditional inorganic salts whose melting points are much higher (> 500 °C) [3, 4]. The explanation for the depletion of the melting point is associated with the size and the non-symmetry of the salt’s components, leading to an unstable crystalline network with a lower lattice energy and, thus, easier to melt [5]. However, the first generation of ILs were unstable and sensitive to water and air [6] and since then, conventionally more ILs have been and are emerging as promising alternatives to conventional organic solvents, as stimuli-responsive indicators [7–10], etc., used in various chemical applications [11], and some are even solid at room temperature, and are denoted as ionic solids (ISs) [12].

Since ILs are salts, the wise pairing of their cations and anions allows them to modulate the final properties of the compound [4, 6, 13–15], offering a wide range of possibilities and applications [16] and these fascinating properties make them attractive for “green” chemistry and sustainable technologies [17–19]. Furthermore, the ability to tailor their properties has led to the design and development of task-specific ILs, designed for specific applications [20]. Despite extensive research on ILs, there is growing interest in exploring their precursors, as their structural analysis can offer insights into the behaviour of ionic compounds [21]. In particular, the chromogenic properties of ILs, which refer to their ability to exist or exhibit colour change properties that arise from several factors, including the presence of specific ions or additives, their ability to interact with dissolved substances, and their response to external stimuli such as light, temperature, or electric fields, have garnered significant attention over the years [2].

The colour change observed in ILs can be attributed to various phenomena, including solvatochromism, thermochromism, photochromism, and electrochromism [22], and the interaction between the dissolved chromophores. In the context of this interesting property, this study explored a series of p-anisidinium ISs that were synthesized using a metathesis method, with p-anisidinium chloride used as a precursor material for these compounds. The obtained products were characterized and existed in different colours, which then prompted the aim of this study which was to evaluate these compounds as alternative materials that can be used as potential DNA-staining dyes.

The reason for this is that it has been previously reported that small organic cations bind duplex DNA mainly by intercalation or groove binding, and the preferred mode is strongly controlled by molecular shape. DNA intercalators usually require a flat, rigid, π-conjugated aromatic surface that can slide between adjacent base pairs and form π–π stacking interactions; the positive charge helps electrostatic attraction to the negatively charged phosphate backbone, but planarity and aromatic surface area are critical for insertion between base pairs [23]. In contrast, minor-groove binders do not need a large flat intercalating surface; instead, they generally require an isohelical or crescent-shaped molecular geometry that matches the curvature of the DNA minor groove, together with cationic groups such as amidinium, guanidinium, ammonium, or related heterocyclic cations for electrostatic attraction, and properly positioned hydrogen-bond donors/acceptors for recognition of groove-edge base-pair patterns [24]. In this context, p-anisidinium-based ionic solids differ from classical DNA intercalators because they are structurally simpler, containing only one substituted benzene ring bearing a methoxy group and a protonated anilinium group and therefore lacks the extended fused planar aromatic surface normally required for strong intercalation [10]. However, their protonated anilinium group can provide cationic character for electrostatic attraction to DNA phosphates, while the methoxy group and counter-anion may influence solubility, hydrogen bonding, and optical response, and this may provide a cost and handling advantage over conventional DNA stains, even though a proven sensitivity advantage is yet to be determined [25].

In contrast, ethidium bromide is still widely used due to its affordability and sufficient sensitivity. However, it is also reported to be mutagenic and is frequently treated as hazardous laboratory waste, which increases the handling and disposal burdens [25]. SYBR Green-type stains are generally more sensitive and safer alternatives to ethidium bromide, but they can alter electrophoretic mobility and DNA size estimation, and some studies also report genotoxicity-related concerns under UV exposure [26]. Conversely, since p-anisidinium-based ISs have been previously confirmed to have structural purity and useful thermal stability [12], this makes them attractive as low-cost, easily handled, tunable solid-state staining candidates, especially because the anion can be modified to tune solubility, fluorescence behaviour, DNA affinity, and background signal. Even though proposed as alternatives, their advantage over ethidium bromide or SYBR Green is still in a ‘potential’ state until direct DNA-staining sensitivity, limit of detection, cytotoxicity, and mutagenicity data is experimentally demonstrated.

The absorption, distribution, metabolism and excretion (ADME) screening of biochemical materials has become increasingly important in identifying and optimizing lead structures. Each ADME process can be further evaluated using certain parameters. For instance, absorption is evaluated mainly using solubility and membrane permeability; distribution using protein binding, tissue binding, and transporters such as P-glycoprotein, which is a key factor in distribution to the central nervous system (CNS); metabolism using metabolic stability in liver microsomes or hepatic clearance; and excretion using renal clearance and urinary excretion rates. Many screening systems have been developed and applied to evaluate compounds based on the appropriate criteria during lead identification and optimization. To streamline these stages, ADME prediction using in silico models has become essential, and is ideally used to extract compounds for high-throughput screening from vast compound libraries or to guide structural design before chemical synthesis [27–29] (de Souza, et al., 2020; Ferreira and Andricopulo, 2019; Komura, et al., 2023). Also, Zheng and co-workers engaged the ADMET prediction model to evaluate the drug-likeness attributes, potential pharmacokinetic behaviors, and toxicity profiles of some newly designed tamoxifen-based ionic solids [30, 31].

Molecular docking offers an efficient alternative by accurately modeling nanoparticle-biomacromolecule interactions at the atomic level, enabling rapid identification of proteins with specific binding affinity to nanoparticles [32]. Following the construction of the initial structure and the selection of a suitable force field, the dynamic process of the nano-bio interface interaction can be simulated and reproduced for detailed investigation based on the corresponding theoretical principle [33, 34]. The density functional theory (DFT) is another computational method widely used to elucidate electronic structures and stability of ionic complexes. The DFT has emerged as a robust method for predicting thermodynamic properties of chemical systems, predicting their solvation energies and reactivity properties [35–37]. Therefore, this study has incorporated in silico ADMET, molecular docking and DFT simulations to complement the experimental synthesis, structural activity relationship and DNA binding potential of the p-anisidinium-based ionic solids.

## Materials and Methods

The chemicals and solvents used in this study were used without further purification and were purchased from Sigma-Aldrich (South Africa). P-anisidinium chloride [p-Anis]^+^[Cl]^−^ (for synthesis), sodium acetate (AR, unhydrous), sodium dodecyl sulfate (GC, BioUltra, for molecular biology, ≥ 99.0%), sodium salicylate (HPLC, ≥ 99.5%), and sodium nitrite (AR), methanol (MeOH, HPLC, GC, LiChrosolv^®^), and water (LC/MS, LiChrosolv^®^). Ethanol (EtOH, AR) and methanol (MeOH, AR) were purchased from Laboquip (KZN, South Africa) NaNO_2_.

### Characterization

The structural elucidation, thermal stability, functional groups, melting point, and chromogenic absorbances were all measured and evaluated using proton nuclear magnetic resonance ^1^H and ^13^C-NMR (Brucker Avance, 600 MHz, UKZN). Approximately 5 mg of material was diluted in 1.5 mL of deuterated dimethylsulfoxide solvent (d6). To obtain ideal, smooth spectra, the chemicals were analyzed using Transform Infrared Spectroscopy (FTIR, Agilent Cary 360, DUT) in the 4000 –650 cm^− 1^ range with a resolution of 4 cm^− 1^ and 120 scans per sample. A dried sample (~ 1 mg) was placed on a metallic ATR stage equipped with a diamond. A Cole-Parmer StuartTM (DUT) electrothermal melting point apparatus (Genesy 50, ThermoFischer, Durban University of Technology, South Africa) equipped with a UV-visible spectrophotometer and a digital thermometer was used to measure the melting points of the compounds. A predetermined volume (1 mL) of the sample was transferred and scanned in the 200–800 nm range.

### Synthesis of ISs

**Scheme 1:**
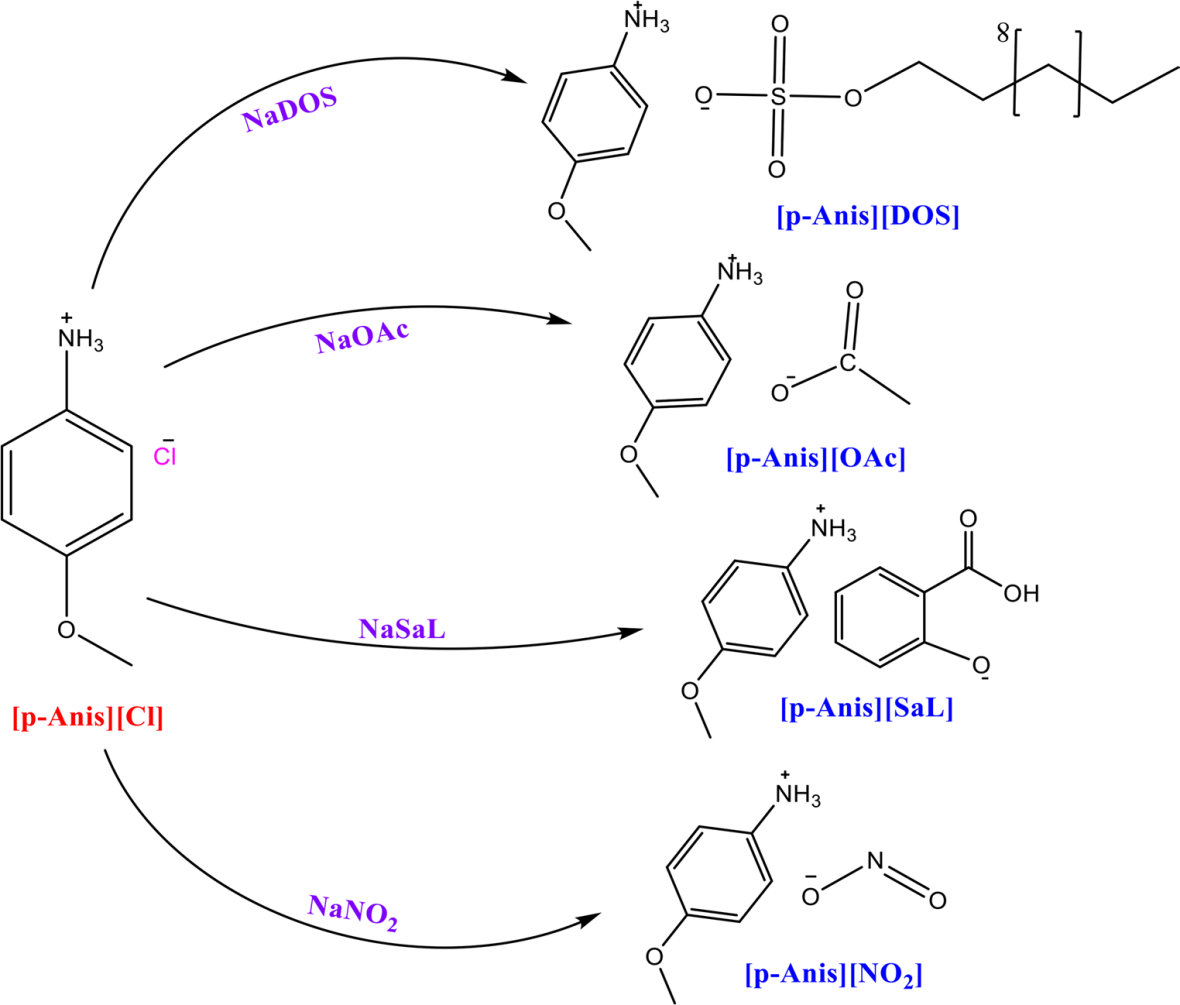
Synthetic pathway of p-anisidinium-based ISs

The ISs were synthesized using a metathesis approach (as per Scheme 1), which was previously reported in our previously published article [12] with guidance from other published studies as well [38, 39]. In this study, two additional compounds, namely: [p-Anis]^+^[DOS]^−^ and [p-Anis]^+^[SaL]^−^ were synthesized, characterized, and their spectroscopic data are reported as supplementary information.

### Anion-Dependent UV-Vis Behavior Studies

To observe the actual colour of each compound, a solvent dissolution approach was formulated with guidance using a review article, which was reported by Santos, Figueirinhas [2], where stock solutions of the ISs were prepared in EtOH, where a required amount of the ionic compound (10 mg) was dissolved in 50 mL of the solvent. After dissolution, electronic spectra scanning was conducted by transferring 1 mL of each solution to a cuvette and scanned within a range of 200–800 nm.

### DNA Staining and Gel Electrophoresis

The compounds were evaluated in two ways, as loading or fluorescence dyes, and with guidance from previously published method [40]. As fluorescence dyes, a 1% agarose gel was prepared in a 1X Tris base, acetic acid, and EDTA (TAE) buffer, and microwaved for 3 min to dissolve. After dissolving, 2 µL of either IS solutions (unknown dyes, 10 mg/mL) or EtBr (known dye, 10 mg/mL) was added to the mixture, cooled to room temperature, poured into a gel tray with a comb, and rested until firm. After solidification, the comb was removed, the gel was placed in a tank filled with buffer solution, and bacterial DNA samples (from 10 ng/ µL, a volume of 3µL from *Escherichia coli- E. coli*, *and Clostridium*) containing a loading dye (2 µL of 6X Tris track DNA, in triplicate) and a reference DNA ladder (duplicate, 1 Kb plus) were loaded into the wells and run across the gel. On the other hand, the compounds were screened as potent loading dyes, with EtBr serving as the fluorescent dye. The DNA samples (3 µL) were mixed with the IS solutions (2 µL), transferred to the wells, and the gel was run for 60 min at 80 volts. After the run, the gel was examined with the naked eye to check whether there were any tracking bands to show the dye’s migration, and then under UV with a Bio-Rad transilluminator to see the fluorescence qualities of the ISs while interacting with DNA.

### Molecular Docking

#### DFT Methods

As per gel electrophoresis results, only the structure of [p-Anis]^+^[NO_2_]^-^ ionic solid was constructed in 2D using ChemDraw professional v15.0 program and then converted to 3D structure using GaussView 6.0 program. The latter program was also used to set the molecule up for DFT calculations and visualization of the output files [37, 41, 42]. The Gaussian 16 vC01 software on the Center for High Performance Computing (CHPC) cluster in Cape Town was used for the calculations. The structure was first optimized to obtain a local-minimum structure and then submitted for single point energy calculation to obtain electronic properties [43]. All calculations were done with the b3lyp functional and 6-311 + + g(d, p) basis set.

#### ADME Methods

The absorption, distribution, metabolism and excretion (ADME) parameters of the ionic solid was predicted using online-based SwissADME program (SwissADME) [44, 45]. Here, the .mol file of the molecule was uploaded to the program’s structure interface and the properties determined by activating the run tab. The SMILE ID of the ionic compound obtained by the program is.

[O-]N = O.[H]C1 = C([H])C(= C([H])C([H])=C1OC([H])([H])[H])[N+]([H])([H])[H].

#### Docking Methods

The crystal structures of proteins corresponding to the experimental bacteria DNA samples were obtained and used for docking [46–48]. The structures were retrieved from the protein databank RCSB PDB: Homepage. PDB IDs: 4E81 and 3SRZ for *Escherichia coli* and *Clostridium difficile*, respectively. Each protein crystal structure was prepared by stripping off the water molecules, co-factors and heteroatoms. Meanwhile, 3D optimized structure of [p-Anis]^+^[NO_2_]^-^ was prepared by converting it to PDB format and used for docking. One FDA approved drug for treating each bacterium, ciprofloxacin and metronidazole, was obtained from the drug bank (Browsing Drugs | DrugBank Online) and used as standard drugs for docking. The docking simulation was done using PyRx virtual screening program.

## Results and Discussion

The synthesized ISs were characterized, and their corresponding spectra results are reported in the supplementary information (Figure S1-S5).

### Anion-Dependent UV-Vis Behavior Studies

**Fig. 1:**
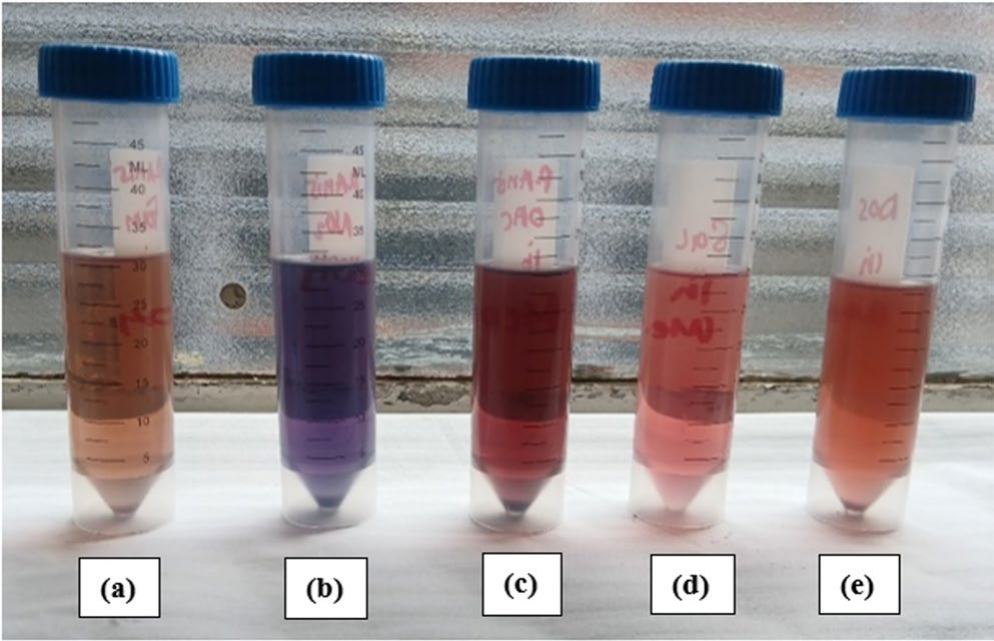
Methanolic solutions of (**a**) Cl^−^, (**b**) NO_2_^−^, (**c**) OAc^−^, (**d**) SaL^−^, and (**e**) DOS^−^ based p-anisidinium ISs [p-Anis]^+^[Cl]^−^

The prepared ISs were intensely dark coloured, and as a result, one would observe and record their actual colours as the same. However, when these compounds were dissolved specifically in methanol, which exhibits good solubility, different chromophores were observed (Fig. 1). These different-colored solutions were scanned to determine the characteristic bands exhibited by compounds that contain a p-anisidine moiety.

**Fig. 2:**
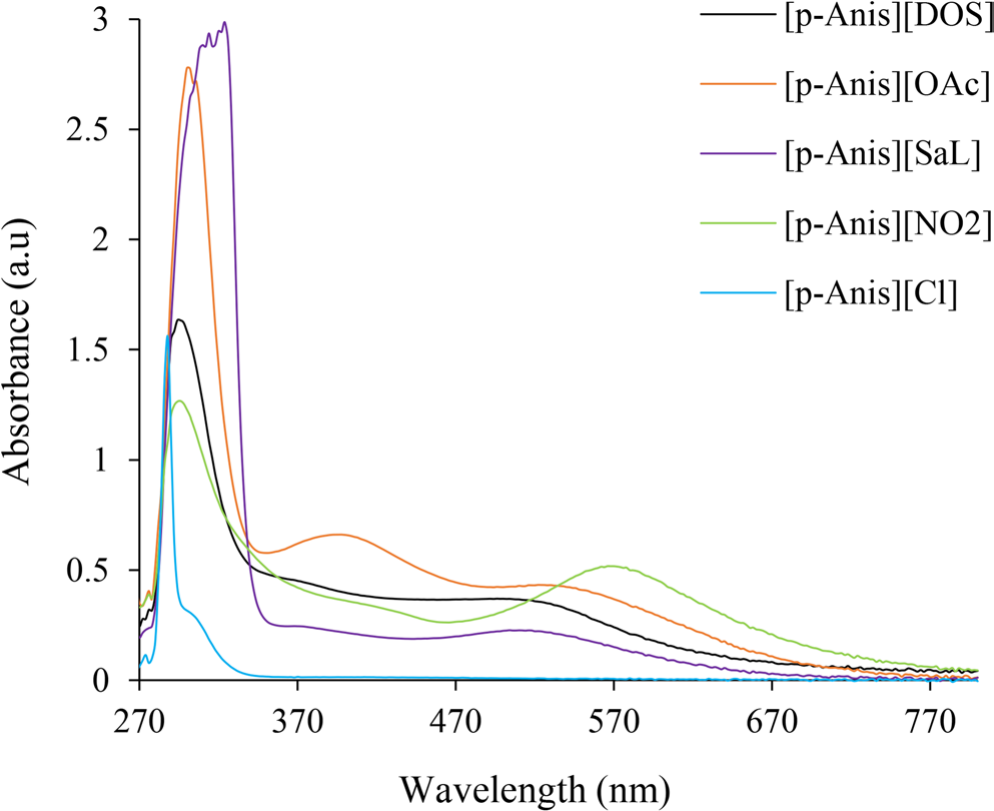
UV-Vis absorption spectra of p-anisidinium based ISs

Like many aromatic compounds, these ISs exhibit characteristic absorption bands in the ultraviolet (UV) region of the electromagnetic spectrum, which arise from the interaction of UV light with the molecule’s electronic structure, specifically the transitions of electrons within the molecule which impact the exact wavelengths based on colour and intensities, and slightly depending on the solvent and anions used [49]. As per Fig. 2, the UV absorbance bands at 270–330 nm are mostly due to permitted π-π* transitions of the aromatic p-anisidinium cation, with additional contributions from the aromatic salicylate anion in [p-Anis][SaL]. Absorption bands at 310 nm (n→π*) for Cl-based, 408 nm (n→π*) for NO_2_-based, 400 nm (n→π*) for OAc-based, 394 nm (n→π*) SaL-based, and 386 nm (n→π*) for DOS-based were observed and attributed to the lone pair on methoxy oxygen (-OCH_3_) and anionic oxygen atoms →π* orbitals of the aromatic ring, carboxylate group -COO^−^, nitrite/nitrate group (NO_2_, NO_3_), sulphate group (^−^O-S).

Particularly for [p-Anis][NO₂], [p-Anis][OAc], [p-Anis][SaL], and [p-Anis][DOS], the low-energy absorption bands (450 –650 nm) observed in the p-anisidinium-based ISs may be tentatively assigned to an intermolecular cation–anion charge-transfer transition rather than a purely intramolecular transition of the p-anisidinium cation. This assignment is supported by the fact that all compounds contain the same p-anisidinium cation but different counter-anions, namely nitrite, nitrate, and acetate, the is evidence of anion-dependent changes in their UV–Vis absorption profile which indicates the involvement of the counter-anion in the electronic transition [12]. Similar UV–Vis-based assignments have been reported for ion-pair charge-transfer salts, where a new red-shifted absorption band relative to the isolated ion precursors was attributed to synergistic cation–anion interactions, and for viologen salts where colour in low-polarity media originates from donor-anion-to-cation charge transfer [50, 51].

**Fig. 3:**
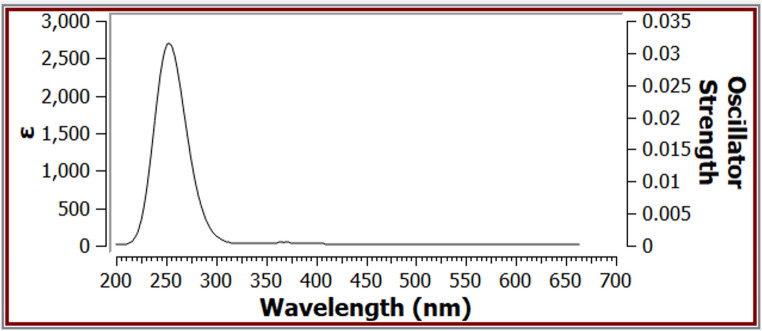
Theoretical UV-Vis absorption spectrum of p-anisidinium based ISs

Conversely, [p-Anis][Cl] has limited apparent charge-transfer character and mostly high-energy π–π* absorption, suggesting a weaker electronic connection between the organic cation and chloride. Compared to pristine material, a blue shift of bands from the corresponding ISs was illustrated, and this is attributed to the electronic interaction between different anions comprised of varied electronegative elements like sulfur and oxygen, with the p-anisidinium cation [52, 53], and the corresponding molar extinction coefficient values are included in the supplementary section. When compared to the theoretical UV-Vis, the NO_2_-based absorbed around the same region (Fig. 3) with the experimentally obtained spectra.

### Effect of Anions on Colour

As well known, p-Anisidine moiety’s chromogenic nature when used as a reagent is based on its ability to produce coloured compounds which stems from its amino group reacting with aldehydes and ketones to form Schiff bases in a condensation reaction, and because of this, this compound is often used in various analytical and industrial applications. The resulting Schiff base possesses an extended conjugated system that allows molecules to absorb light in the visible region, a carbon-nitrogen double bond (C = N), and aromatic ring, which are crucial for the colour change. The more extensive the conjugation, the longer the wavelength of light absorbed, and thus, the more likely the compound is to appear coloured to the human eye. Each colour produced is influenced by the type of aldehyde or ketone used and the resulting structure.

In this case, the prepared ISs consists of an aniline moiety with a methoxy group (-OCH_3_) attached to the aromatic ring. Because of this aniline moiety, p-anisidinium cation (existing as p-anisidine) has been reported for several industrial applications, like in the textile industry as an additive in dye manufacturing [54]. Based on the above analogy, it is quite evident that not only Schiff bases produced can cause colour change, but also different anions that interact with p-anisidinium cation can lead to the production of different chromophore ISs compared. These findings and properties make this class of compounds valuable tools, especially in dye synthesis for various analytical and industrial applications.

### Loading VS Fluorescence Dye

p-anisidinium based ISs were synthesized, characterized, and tested for their ability to bind to DNA. This interest arose from the fact that these compounds existed in different colours (as per observed solvochromism results), and this observation can be attributed to the compound having the aniline framework with a methoxy substituent and is well known to find applications in the textile industry primarily as a starting material or an intermediate in the synthesis of certain dyes and pigments with different vibrant and colourfast shades, including specific hues like Acid Red [55].

**Fig. 4:**
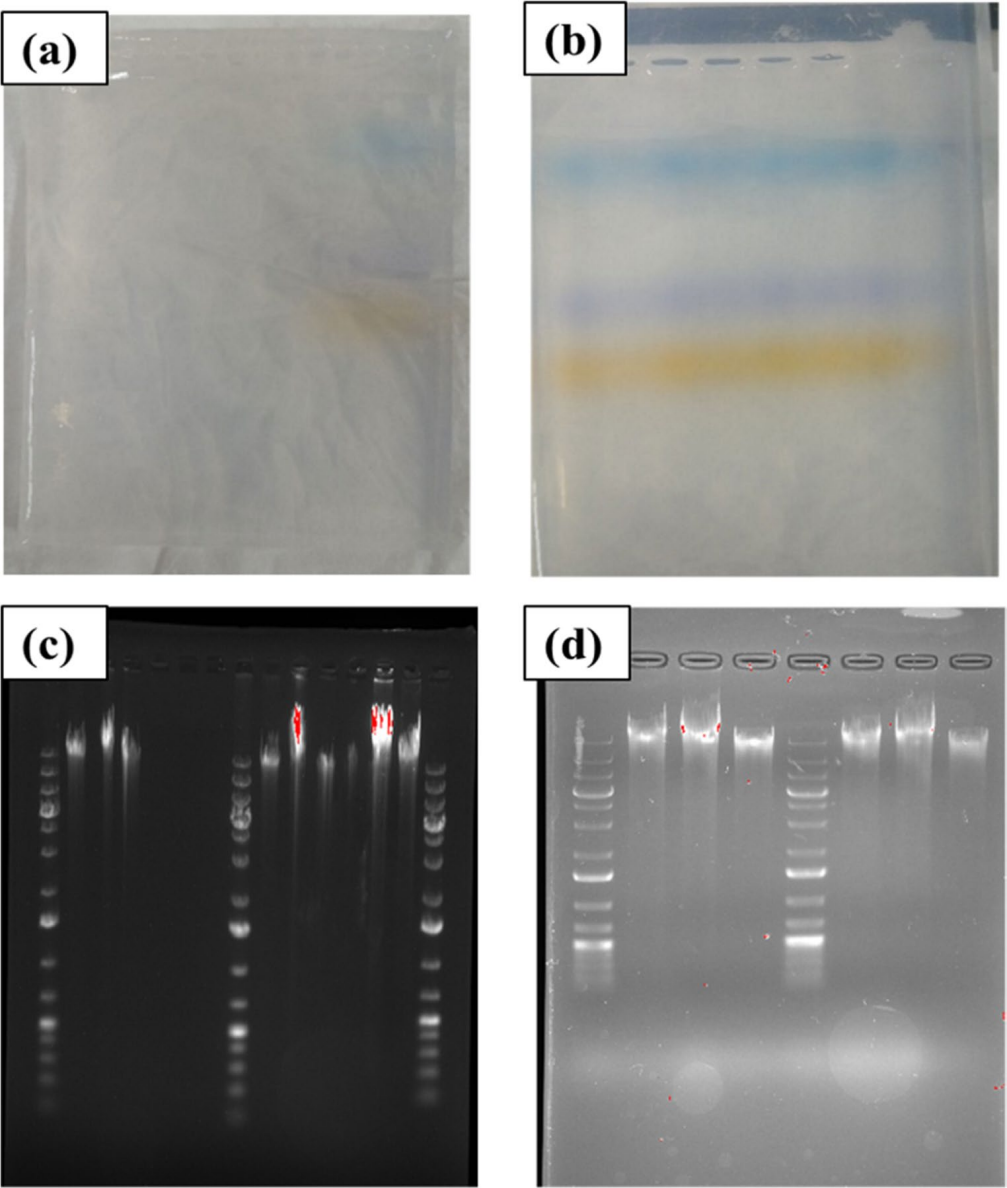
Agarose gel after electrophoresis run with (**a**) Cl^−^, NO_2_^−^, OAc^−^, and SaL^−^ based ISs used as loading dyes, (**b**) with NO_2_^−^ based only among the examined used as a fluorescence dye, (**c**) agarose gel from NO_2_^−^ based as a loading dye under UV showing DNA bands, and (**d**) agarose gel from NO_2_^−^ based as a fluorescence dye under UV showing DNA bands

After each gel electrophoresis run, the agarose gel illustrated as Fig. 4 (a) showed different coloured migration bands (blue, purple and orange) only for the DNA ladder and faintly for [p-Anis]^+^[NO_2_]^-^ IS used as a loading dye, while in Fig. 4 (b), migration bands for all loaded DNA samples with [p-Anis]^+^[NO_2_]^-^being used as a fluorescence dye, were visible. These observed colours were attributed to the fact that most loading dyes are intentionally chosen to primarily serve as coloured ionic indicators that facilitate sample loading and electrophoretic monitoring because they have different molecular sizes and charges, causing them to migrate through the agarose gel at different rates, and generating different colours as they intercalate with DNA. Conversely, when observed under UV light, both agarose gels illustrated as Fig. 4 (c) and (d) showed fluorescence bands at the same position corresponding to the DNA fragments with reference from the ladder. This illustrated that [p-Anis]^+^[NO_2_]^-^ has the potential to be used as both loading and fluorescence dye but with certain limitations that when used as a loading dye, the tracking bands are faint and it becomes difficult to clearly check the migration pattern of the dye, and if it is migrating correctly. The better staining performance of [p-Anis]^-^[NO_2_]^+^ amongst the examined may be attributed to the combined effects of stronger ion-pair-assisted optical response, reduced aggregation, favourable solubility, and improved accessibility for DNA interaction [12, 56, 57], and this observation may also be related to its distinct optical properties. Unlike conventional tracking dyes, which often contain extensive long-chain conjugated systems, [p-Anis]^-^[NO_2_]^+^ lacks extensive conjugation, which may be attributed to the appearance of relatively faint bands during electrophoresis. In addition, the NO_2_^-^ anion may act as a fluorescence quencher, further contributing to the observed weak colouration [58]. These results then suggest that this compound may operate through mechanism with the gel matrix that differs from that of ethidium bromide EtBr.

Comparing the fluorescence patterns of DNA in the presence and absence of [p-Anis]^+^[NO_2_]^-^, and against EtBr and a loading dye, it is evident that this IS can bind with DNA and provide augmented fluorescence when exposed under UV and from this observation, the compound can potentially be used as an alternative fluorescence dye, more than a loading dye. The reason being, mixing DNA samples with [p-Anis]^+^[NO_2_]^-^ solution was challenging because the mixed solution became faint and was almost unnoticeable when loaded on the wells, almost colourless. This made loading the samples to the wells challenging under a naked eye, but after 1 h of running the electrophoresis and viewing under UV, DNA fragment bands corresponding to that of the DNA ladder were observed.

### Quantum Chemical Descriptors

The quantum chemical descriptors for the ionic solid are presented Table 1. Meanwhile, the fundamental parameters obtained from electronic structure calculations are LUMO and HOMO energies known as lowest unoccupied and highest occupied molecular orbitals, respectively [35–37]. These parameters were used to determine other descriptors according to the Eqs. (1)–(8).

**Table 1:** Calculated quantum chemical descriptors

	E_LUMO_	E_HOMO_	ΔE	I	A	η	δ	χ	C_p_	ω	µ_D_
[p-Anis]^+^[NO_2_]^−^	-1.70	-5.11	3.40	5.11	1.70	1.70	0.58	3.41	-3.41	3.41	1.12

All units are in eV except µ_D_ (Debye). ΔE: Energy gap A: Electron affinity, I: Ionization potential, η: Global hardness, δ: Global softness, χ: Electronegativity, C_p_: Electronic chemical potential, ω: Global electrophilicity, µ_D_: Dipole moment

1 $$\:\varDelta\:E={E}_{LUMO}-\:{E}_{HOMO}$$

2 $$\:I=\:-{E}_{HOMO}$$

3 $$\:A=\:{-E}_{LUMO}$$

4 $$\:\eta\:=\:\frac{\varDelta\:E}{2}$$

5 $$\:\delta\:=\:\frac{1}{\eta\:}$$

6 $$\:\chi\:=\:\frac{(I+A)}{2}$$

7 $$\:{C}_{P}=\:-\chi\:$$

8 $$\:\omega\:=\:\frac{{\chi\:}^{2}}{\varDelta\:E}$$

The conceptual density functional theory (DFT), otherwise known as density functional reactivity theory, is an undertaken in literature to elucidate molecular properties related to stability and reactivity [59–61]. The difference between the LUMO and HOMO energy (Eq. 1) is the energy gap. Energy gap (∆E) is a measure of the chemical reactivity of a molecule in which case, the lower the energy gap, the greater the chemical reactivity and vice versa. Here, the band gap 3.4 eV is comparable to that of semiconductors [62] and it demonstrates reasonable electronic conductivity. These can be attributed to delocalized electrons in the benzene ring and the ionic nature of the neighboring nitro group (Table 2).

**Table 2:** Calculated HOMO-LUMO energy gap and experimental wavelengths (λ_max_)

	[*p*-Anis]^+^[SaL]^−^	[*p*-Anis]^+^[ NO_2_]^−^	[*p*-Anis]^+^[DOS]^−^	[*p*-Anis]^+^[OAc]^−^	[*p*-Anis]^+^[Cl]^−^
∆E	3.38	3.40	4.92	5.12	5.17
π→π*	326	289	290	306	288
CTT	522	574	512	538	–

Within the Kohn-Sham (KS) framework of DFT, the highest occupied KS orbital energy is the negative of the exact ionization potential (Eq. 2) while the lowest unoccupied KS orbital energy is the negative of the electron affinity (Eq. 3) [63, 64]. Both being negative of the exact data obtained from DFT calculations, they are also referred to as fundamental parameters and can be used to determine other chemical quantities [65, 66].

Global hardness (η) is a quantity that determines a molecule’s susceptibility to charge transfer. It is directly related to energy gap (∆E) but inversely related to the global softness (δ) [67, 68]. Here, the η value 1.70 eV is relatively low, and it indicates that the ionic solid is chemically reactive. Meanwhile, electronegativity (χ) measures the electron distribution in a molecule and the molecule’s propensity to accept electrons [69]. This can be attributed to first, the electron-withdrawing nature of the nitro group, and secondly the resonance effects on both the nitro group and benzene ring.

According to Parr and Pearson [70], the electronic chemical potential describes the escaping tendency of electron from a stable system. It is a thermodynamic quantity which describes the change in a molecule’s energy when a particle is added or removed, while other related variables are fixed [71, 72]. Here, the ionic solid has electronic chemical potential of -3.41 eV indicating a tendency to lose electrons and attain more stable state. The global electrophilicity index (ꞷ) is a quantity that describes the stabilization energy of a molecule when saturated by electrons approaching from the surroundings [69, 73]. A low ꞷ value of 3.41 eV value obtained for the ionic solid indicates low stabilization energy, high susceptibility to charge transfer and increasing chemical reactivity.

Dipole moment (µ_D_) determines the overall polarity of a system and enhances evaluation of its percentage ionic character arising from differences in the electronegativity of the bonding atoms [74, 75]. The dipole moment of the ionic solid 1.12 D is low, and it indicates small differences in the electronegativity of the atoms. This can be attributed to the fact that though the nitro group is electronegative, the cyclic aromatic group has electronegative amino and methoxy substituents.

**Fig. 5:**
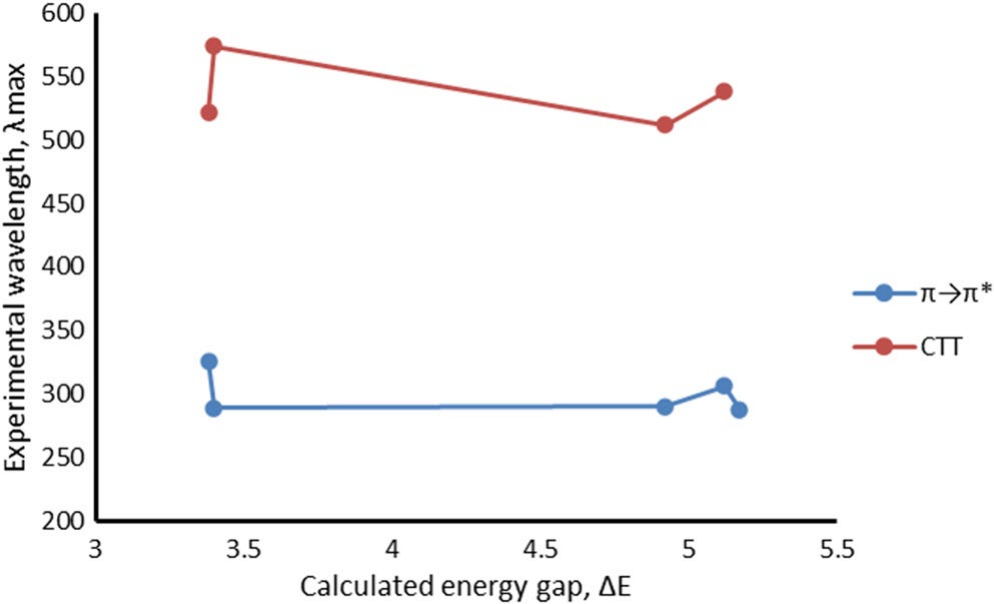
A plot of experimental wavelengths from π→π* and CTT transitions versus calculated energy gaps

A correlation is observed between the observed/experimental wavelength and the calculated HOMO-LUMO energy gap (Table 2). Here, the data for all allowed transitions, π→π* and CTT, are reported. The data for these are compiled and presented in Table 2. A plot of the data is presented in Fig. 5. From the plot, there is a fluctuating relationship between the wavelength from allowed transition and the energy gap. For π→π*, the wavelength increases, then decreases and increases again as the energy gap keeps increasing. In CTT, the wavelength decreases, then increases and then decreases again as the energy gap continues to increase.

### Frontier Molecular Orbitals and Molecular Electrostatic Potential Maps

**Fig. 6:**
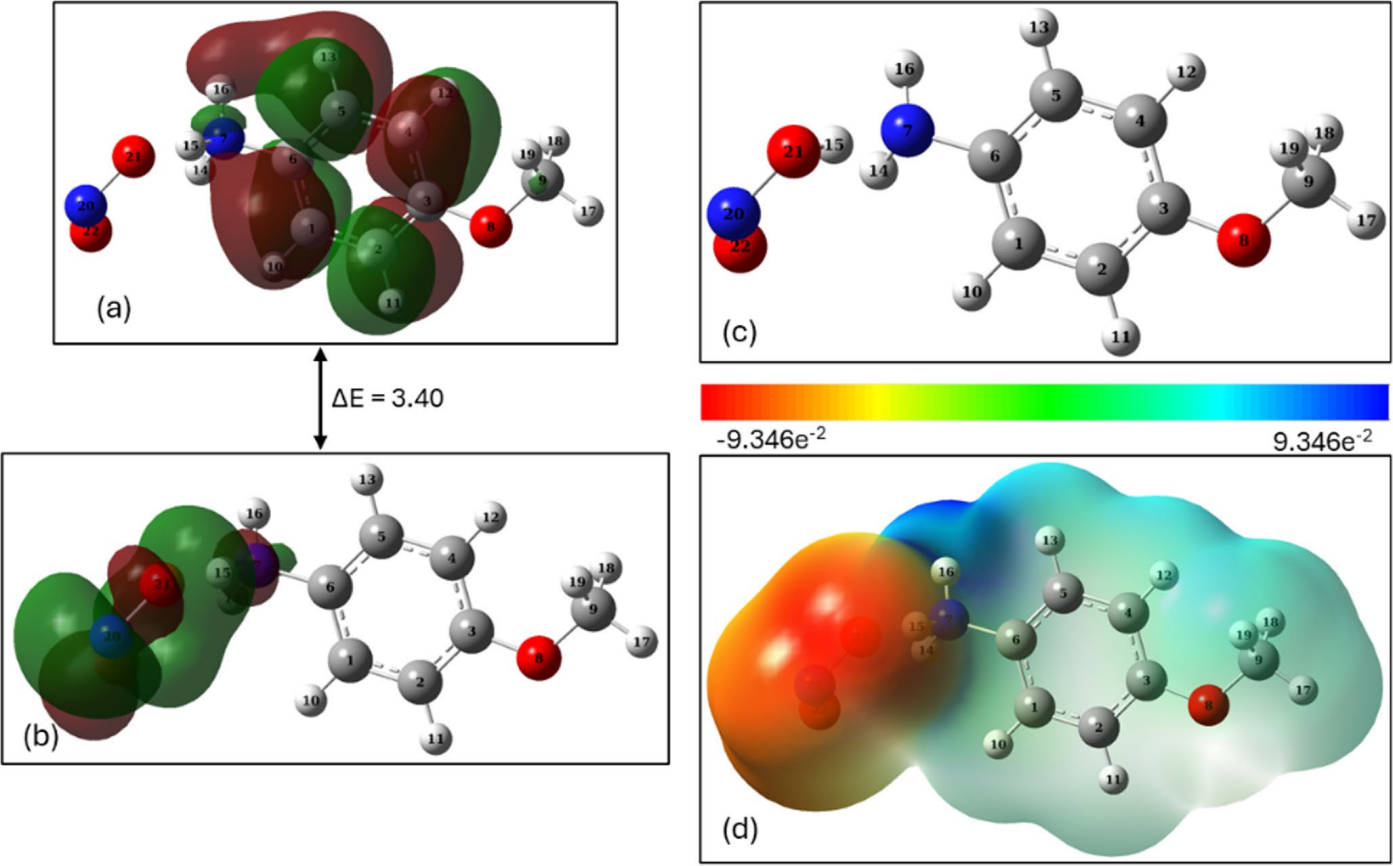
(**a**) LUMO and (**b**) HOMO orbitals, (**c**) optimized structure and (**d**) MEP map of [p-Anis]^+^[NO_2_]^−^

The frontier molecular orbitals obtained for the ionic solid are presented in Fig. 6a and b. In the LUMO (Fig. 6a) there are contributions from aromatic carbons and hydrogen. Also, there is contribution from one of the amine hydrogen atoms. The HOMO, on the other hand, receives contributions from the nitro group, and the amine hydrogen directed towards the nitro group. The optimized structure of the ionic solid is presented in Fig. 6c.

The molecular electrostatic potential map (MEP) is a pictorial description of the charge distribution across a molecule. It helps to understand the drug-receptor interactions, biological recognition process and chemical reactivity potential of molecules [76, 77]. Generally, in a MEP map, the red region is one which is electron rich, of negative electrostatic potential and is prone to electrophilic attack. On the other hand, the blue region is one which is electron deficient, of positive potential and prone to nucleophilic attack [76, 78]. The MEP obtained for the ionic solid is presented in Fig. 1d. Here, the blue region is dominantly on the amine hydrogen atoms, suggesting sites of nucleophilic attack while the red region is around the nitro group suggesting sites of electrophilic attack. Thus, the amine substituent of the p-anisidinium is the most electrophilic region and would potentially interact with the nucleophilic sites of DNA. Similarly, nitro group is the most nucleophilic region and would potentially interact with the electrophilic sites of DNA.

### ADME Properties Analysis

The absorption, distribution, metabolism and elimination (ADME) properties prediction of chemical compounds has attracted considerate research interest in recent years because it increases the success rate of compounds reaching development [79]. The ADME parameters obtained for the ionic solid are presented in Table 3.

**Table 3:** ADME Parameters

Properties	[*p*-Anis]^+^[NO_2_]^−^
Formula	C_7_H_10_N_2_O_3_
Molecular Weight (gmol^− 1^)	170.17
No. of heavy atoms	12
Fraction Csp3	0.14
No. of rotatable bond	1
No. of H-bond acceptors	5
No. of H-bond donors	1
Molar refractivity	46.78
TPSA (Å^2^)	89.36
MlogP	-3.09
Solubility class	Soluble
GI absorption	High
BBB permeability	No
Lipinski’s rule of 5	Yes (0 violation)
Verber rule	Yes
Bioavailability score	0.55

The fraction Csp3 quantifies the number of sp3 hybrid carbons to the total carbons in a molecule, and it is expected in the range 0.25 to 1.00 for saturation and penetration through the cell membrane [80]. Here, the ionic solid has fraction Csp3 0.14, indicating poor solubility in lipid membrane and inability to permeate the cell membrane.

Lipinski’s rule of 5 (Ro5) is rule of thumb for evaluating drug likeness i.e. whether a chemical compound would be a promising orally active drug candidate in humans or not [81]. The rule of thumb as implemented by Lipinski and incorporated in SwissADME states that acceptable range of parameters are; molecular weight between 150 and 500 g/mol, MLogP (lipophilicity) below 5, number of hydrogen bond donors ≤ 5, number hydrogen bond acceptor ≤ 10 [82, 83], . Similarly, the Verber’s rule [84, 85] of drug likeness sets range of TPSA between 20 and 130 Å and rotatable bond (flexibility) between 1 and 9. As seen in Table 2, the ionic solid has values for the parameters within acceptable ranges with no violation of either Lipinski’s Ro5 or Verber’s rule. This informs that the compound has promising drug likeness and could possibly be an orally active drug candidate [74].

Furthermore, a bioavailability score of 0.55 or higher is generally considered good, particularly for orally administered drugs [86]. Here, the ionic solid has bioavailability score of 0.55, suggesting that it could have good candidate for oral bioavailability study. In other words, a significant amount of the compound, if delivered as drug trial, will successfully reach the systemic circulation in its active form.

**Fig. 7:**
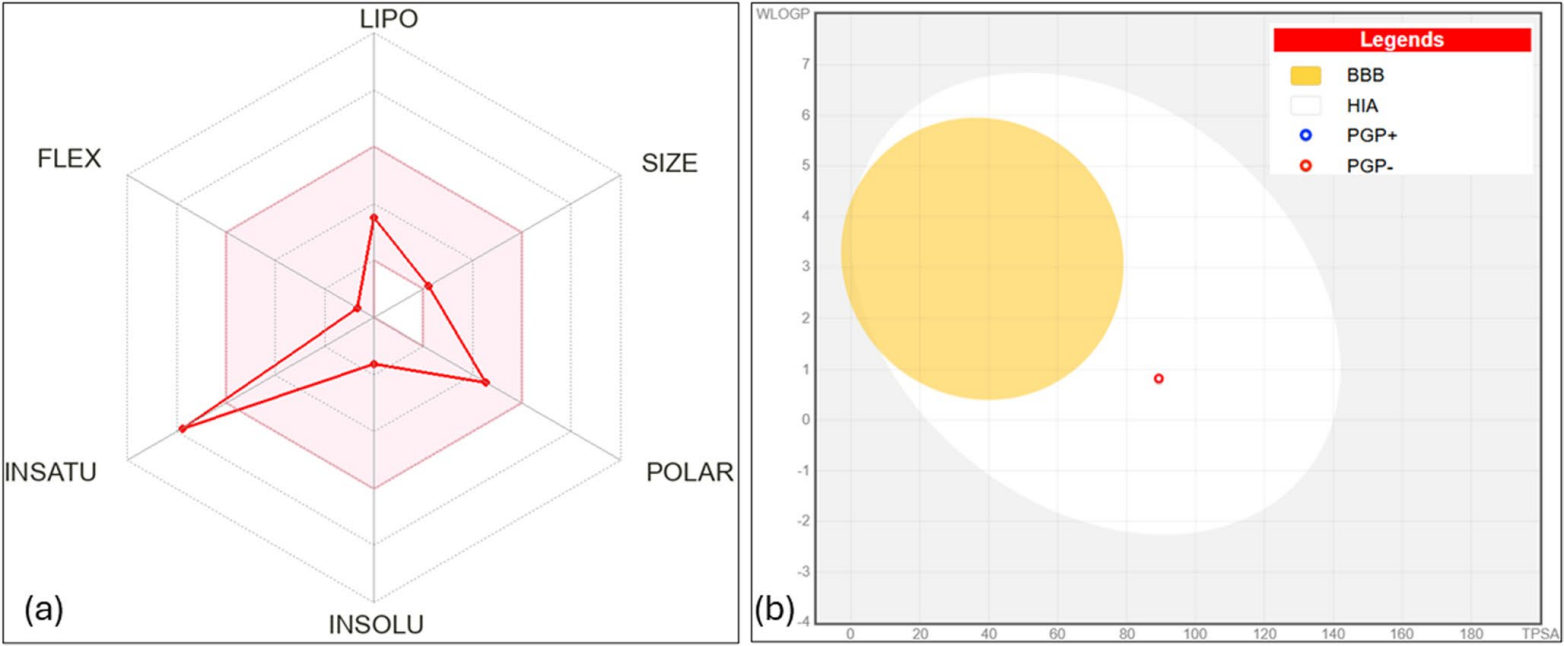
(**a**) The BOILED-Egg model and (**b**) bioavailability radar plot of [p-Anis]^+^[NO_2_]^−^

Some ADME parameters are presented in Table 3, and the corresponding diagrams obtained for the ionic solid are presented in Fig. 7. Figure 7a is the radar plot which showcases the ideal zone for all attributes i.e. acceptable range of parameters. For each parameter, the zone within the pink area is the acceptable range while zone outside the pink area is out of acceptable range. Here, only the insaturation (INSATU), a description of the fraction Csp3 is out of acceptable range while other fours parameters; flexibility (FLEXI), lipophilicity (LIPO), molecular weight (SIZE), polarity (POLAR) and insolubility (INSOLU) are within the acceptable range [87].

Figure 7b is the BOILED-Egg model, an intuitive graphical classification model for gastrointestinal absorption and brain access. Here, the ionic solid molecule is indicated by a red dot suggesting that the molecule is not effluated from the central nervous system by the P-glycoprotein [45]. Also, it is in the BOILED-Egg’s white suggesting that it could make passive absorption by the gastrointestinal tract.

### Molecular Docking Analysis

Molecular docking is a key tool in structural molecular biology and computer-aided drug design with the goal of predicting the predominant binding modes of a ligand with protein and using a scoring function to correctly rank the candidate molecules [88]. Here, [p-Anis]^+^[NO_2_]^−^ and the standard drugs (ligands) were docked against experimental bacterial DNA. The binding energy for each ligand-protein pair is presented in Table 4.

**Table 4:** Binding energies

	Ciprofloxacin	Metronidazole	[*p*-Anis]^+^[NO_2_]^−^
*E. Coli (kcal/mol)*	-6.9	-	-4.9
*C. Difficile* (kcal/mol)	-	-5.7	-5.9

For the gram-negative bacterium *Escherichia coli*, ciprofloxacin has binding energy − 6.9 kcal/mol while [p-Anis]^+^[NO_2_]^−^ has − 4.9 kcal/mol. These suggest that the standard drug binds more strongly to the protein and would perform better as antibacterial than the ionic solid. Nevertheless, binding energy of the ionic solid suggests that the compound has good binding affinity and could be a potential antibacterial to address *E. Coli*.

**Fig. 8:**
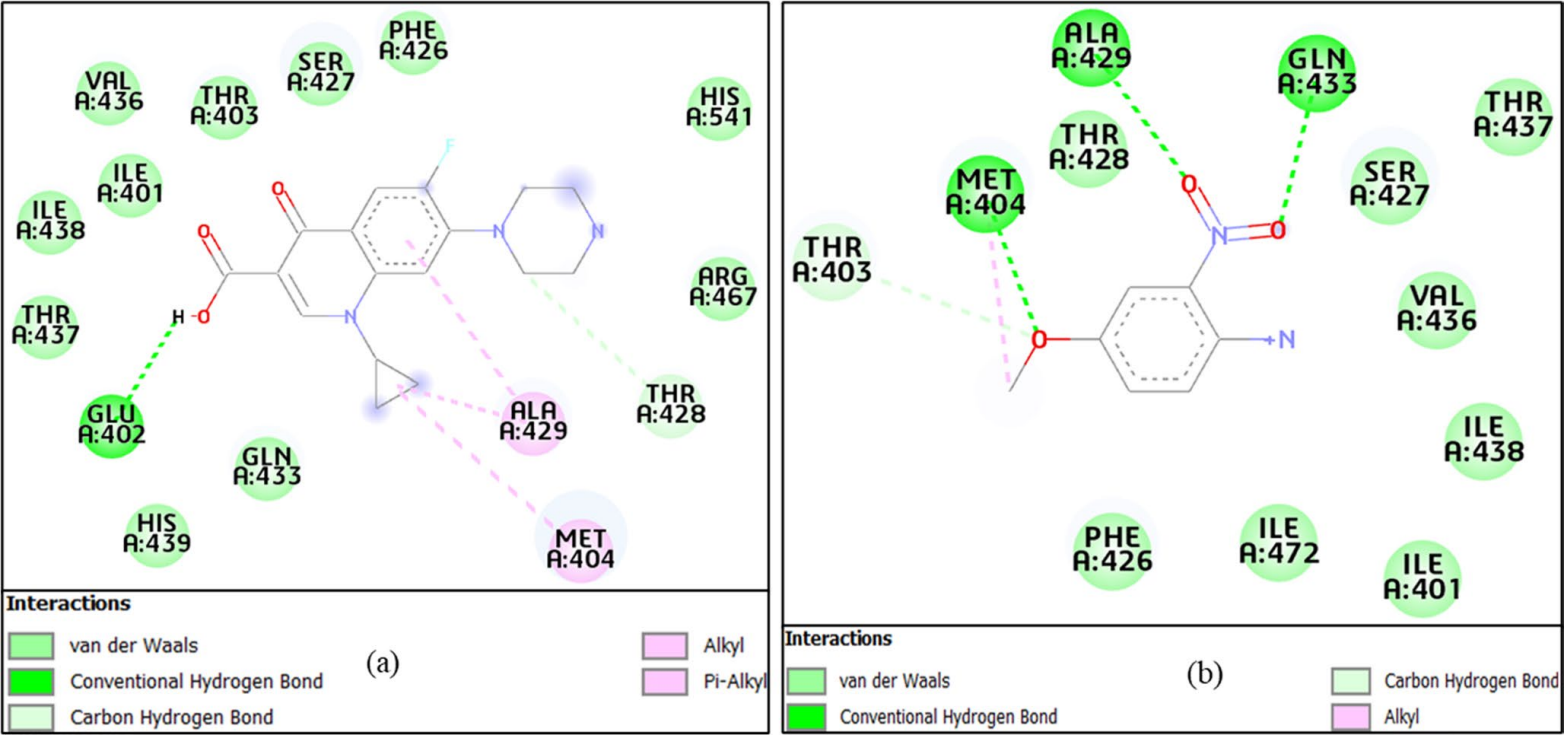
2-dimensional diagram of interactions between toxins of *E. Coli* protein (PDB ID: 4E81) and (**a**) ciprofloxacin (**b**) [p-Anis]^+^[NO_2_]^−^

**Fig. 9:**
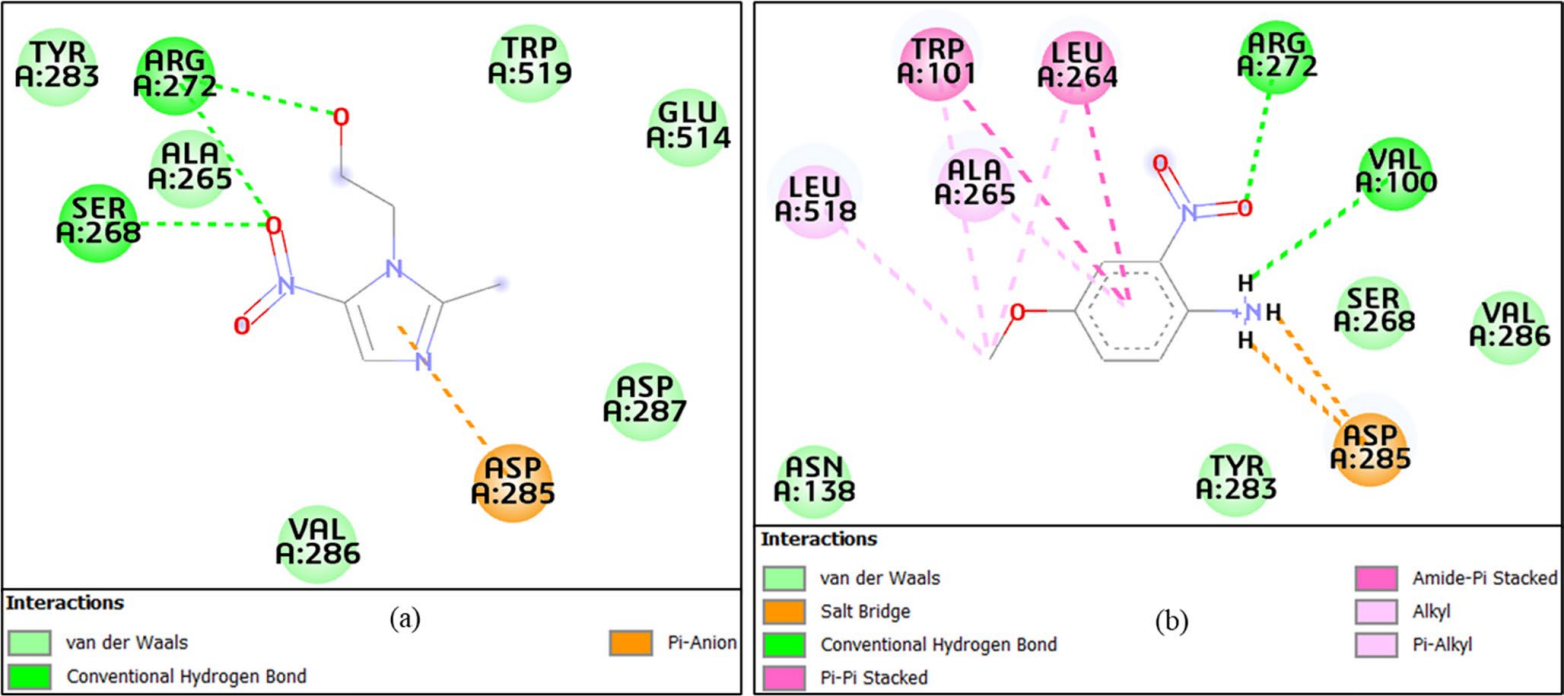
2-dimensional diagram of interactions between toxins of *C. difficile* A (TcdA) protein (PDB ID: 3SRZ) and (**a**) metronidazole (**b**) [p-Anis]^+^[NO_2_]^−^

For the gram-positive bacterium *Clostridium difficile*, metronidazole has binding energy − 5.7 kcal/mol while [p-Anis]^+^[NO_2_]^−^ has − 5.9 kcal/mol. These suggest that both the standard drug and the ionic solid have same binding potential to the protein target and would strongly inhibit the disease in the potential. Meanwhile, the 0.2 kcal/mol binding energy difference suggests that the ionic solid could binds more strongly and potentially be a better antibacterial against *Clostridium difficile* than metronidazole. The ionic solid has lower binding energy (-5.9 kcal/mol) in *C. difficile* than in *E. coli* (-4.9 kcal/mol), indicating higher potential in the former. The 2-dimensional diagrams of the binding interactions are presented in Figs. 8 and 9.

In Ciprofloxacin–4E81 complex (Fig. 8a), the key interacting residues are Glu402 with conventional hydrogen bond, Thr428 with carbon hydrogen bond, Ala429 with alkyl and Met404 with pi-alkyl interactions. For [p-Anis]^+^[NO_2_]^−^–4E81 complex (Fig. 8b), the key interacting residues are Met404 with both conventional hydrogen bond and alkyl interactions, Thr403 with carbon hydrogen bond and both Ala429 and Gln433 with conventional hydrogen bond to each of the nitro oxygen atoms. Other residues are bound to the proteins through van der Waals interactions.

In metronidazole–3SRZ complex (Fig. 9a), key interacting residues are Asp285 with pi-anion interaction to the imidazole ring, Ser268 with conventional hydrogen bond to a nitro oxygen, and Arg272 with conventional hydrogen bond to a nitro oxygen and a hydroxyl oxygen. In [p-Anis]^+^[NO_2_]^−^–3SRZ complex (Fig. 9b), the key residues are Leu518 with alkyl interactions, Ala265 with pi-alkyl interaction, Trp101 with both pi-alkyl and amide-pi stacked interactions and Leu264 with both alkyl and pi-pi stacked interactions. Other key residues include Arg272 and Val100, both with conventional hydrogen bond and Asp285 with salt bridges to two amine hydrogen atoms.

## Conclusion

In continuation from our previously published work, in this study two more p-anisidium-based ISs namely [p-Anis]^+^[DOS]^−^and [p-Anis]^+^[SaL]^−^ were successfully synthesized and characterized and in total all four (including NO_2_^−^ and OAc^−^ based ISs) were evaluated as potential DNA staining materials based on the resultant product colours observed after synthesizing them. Using agarose gel electrophoresis technique, DNA staining was explored with reference to EtBr, and among the examined, different strong coloured migration bands (blue, purple, and orange) only for the DNA ladder and faintly for [p-Anis]^+^[NO_2_]^−^ IS were observed, while under UV these bands suggested fluorescence dye potential with strong migration bands for all loaded DNA samples. Further validation of this observation was done using DFT simulation, and the results suggested that [p-Anis]^+^[NO_2_]^−^ IS would be chemically reactive, with low stabilization energy, high susceptibility to charge transfer, and moderate electrical conductivity. The ADME property prediction shows that the ionic compound obeys both Lipinski and Verber rules suggesting good drug likeness and good oral bioavailability. The BOILED-Egg model suggests that it is not affiliated with the central nervous system by the P-glycoprotein, but it can be passively absorbed by the gastrointestinal tract. Correlating all these findings, the observed molecular docking results to that of gel electrophoresis, it is evident from the obtained binding energies that the is a qualitative relationship between the ability for this IS with DNA to generate migration bars with the obtained binding energies, and the predicted interaction between the DNA and the [p-Anis]^+^[NO_2_]^−^ IS corroborate the observations, also suggesting that the compound has a promising antibacterial activity against both DNA bacteria *Escherichia coli* and *Clostridium difficile*. Future studies on the in-depth mechanism between the DNA-binding and the ISs are required, the sensitivity tests, concentration-dependant staining, and cytotoxicity comparisons studies are required to fully understand the mechanistic process of this interaction.

## Electronic Supplementary Material

Below is the link to the electronic supplementary material.

Supplementary File 1

## Data Availability

All data are presented in the manuscript and in the supplementary section.
