# Supplementary material for: Preliminary Assessment of p-Anisidinium-Based Ionic Solids as Potential DNA Staining Agents: DFT and Molecular Docking Studies
Source: J Fluoresc. 2026 Jul 21;36(7):4793–807. doi: 10.1007/s10895-026-04872-8 (PMC13486037; doi:10.1007/s10895-026-04872-8)
Supplement: Supplementary file 1 [file 10895_2026_4872_MOESM1_ESM.docx]

**Preliminary Assessment of p-Anisidinium-Based Ionic Solids as Potential DNA Staining Agents: DFT and Molecular Docking Studies**

Vuyolwethu Tokoyi^a*^, Eric Oluwafisayo Akintemi^b^, Hadley S. Clayton^b^

and Nirmala Deenadayalu^a^

*^a^ Department of Chemistry, Faculty of Applied Sciences, Durban University of Technology, South Africa*

*^b^ Department of Chemistry, University of South Africa, Florida Science Campus, Johannesburg, 1709, South Africa*

*Corresponding author*: [vuyolwethut@dut.ac.za](mailto:vuyolwethut@dut.ac.za)

# **Supplementary information**

## ***S1: Method***

A vacuum-dried 250 mL round bottom flask (RBF) was filled with the necessary quantity of a pre-weighed [p-Anis]^+^[Cl]^-^ (0.3 mol). After adding 150 mL of ultrapure water and thorough mixing, 0.3 mol of the necessary amount of NaSaL or NaDOS was added to the flask. Overnight, the mixture was kept at room temperature while thin-layered chromatography (TLC) was used to monitor it through to completion. After finishing, the reaction mixture was dried in an oven set at 30 ^o^C for the whole night after being rotary evaporated to decrease solvent. To get rid of the sodium chloride that formed, the acquired crude sample was further cleaned with water and filtered.

## ***S2: Spectroscopic data***

Table S1: Summary table for yields and melting points

| **ISs** | **Yield (%)** | **Melting point (^o^C)** |
| --- | --- | --- |
| [p-Anis]^+^[NO_2_]^-^ | 87 | 147 |
| [p-Anis]^+^[SaL]^-^ | 81 | 105 |
| [p-Anis]^+^[OAc]^-^ | 90 | 97 |
| [p-Anis]^+^[DOS]^-^ | 86 | 123 |

Table S2: Molar extinction coefficients (L mol^-1^cm^-1^) for the synthesized compounds

| Absorption bands | [p-Anis]^+^[DOS]^-^ | [p-Anis]^+^[SaL]^-^ | [p-Anis]^+^[NO_2_]^-^ | [p-Anis]^+^[OAc]^-^ | [p-Anis]^+^[Cl]^-^ |
| --- | --- | --- | --- | --- | --- |
| π→π* | 5.37x10^3^ | 9.83x10^3^ | 4.17x10^3^ | 9.30x10^3^ | 5.20x10^3^ |
| n→π* | 1.37x10^3^ | 8.00x10^2^ | 1.17x10^3^ | 2.23x10^3^ | 8.00x10^3^ |
| Intermolecular cation-anion CTT | 5.33x10^3^ | 7.33x10^2^ | 1.70x10^3^ | 1.50x10^3^ | - |

**Figure S1**: ^1^H-NMR (a) and ^13^C-NMR of p-anisidinium salicylate [p-Anis]^+^[SaL]^-^

**State**: Brown solid. **Yield:** 81% ; Melting point:, 105 °C; FTIR (νmax/cm−1): 2987 (-NH_3_), 1733 (C=C), 1569 (C-N), 1382 (C-O); ^1^H-NMR (400 MHz, CDCl_3_): δ 3.7 (s, 3H), 6.6 (*j* = 8.2 Hz, 2H), 6.7 (*j* = 7.8 Hz, 3H), 6.8 (*j* = 7.6 Hz, 1H), 7.3 (*j* =7.8, 1H), 7.7 (1H), 7.9 (3H); ^13^C-NMR (400 MHz, CDCl_3_): δ 55.7 (a), 113.6 (b), 115.0 (c), 117.4 (d), 119.1 (e), 119.4 (f), 130.8 (g), 134.1 (h), 135.6 (i), 155.3 (j), 161.8 (k), and 174.5 (l); Water content: 4.3 %

**Figure S2**: ^1^H-NMR (a) and ^13^C-NMR of p-anisidinium dodecylsulfate [p-Anis]^+^[DOS]^-^

**State**: Dark maroon solid; Yield: 86 %; Melting point:, 123 °C; FTIR (νmax/cm−1): 2987 (-NH_3_), 1733 (C=C), 1569 (C-N), 1382 (C-O); ^1^H-NMR (400 MHz, CDCl_3_): δ 0.79 (3H), 1.17 (16H), 1.44 (2H), 3.67 (3H), 3.77 (*j* = 7.1 Hz, 2H), 6.74 (*j* = 8.8 Hz, 2H), 7.33 (*j* = 8.8 Hz, 2H), and 9.28 (3H); ^13^C-NMR (400 MHz, CDCl_3_): δ 14.1 (a), 22.7 (b), 25.7 (c), 29.3-29.7 (d), 31.9 (e), 32.7 (f), 55.4 (g), 62.9 (h), 68.9 (i), 114.7 (j), 124.7 (k), and 159.6 (l); Water content: 7.5 %.

Transmittance (%)

| Wavenumber (cm-3) | [p-Anis][Cl] | [p-Anis][DOS] | [p-Anis][SaL] |
| --- | --- | --- | --- |
| 398.825 | 60.3309 | 66.1264 | 59.5279 |
| 402.553 | 76.3724 | 76.7847 | 70.153 |
| 406.28 | 87.7128 | 72.9281 | 65.1146 |
| 410.007 | 84.9539 | 76.2041 | 70.5531 |
| 413.735 | 86.305 | 77.0299 | 57.8529 |
| 417.462 | 80.3136 | 84.7199 | 67.8902 |
| 421.189 | 71.1268 | 78.5283 | 68.1268 |
| 424.916 | 84.3107 | 72.4263 | 86.9696 |
| 428.644 | 80.9174 | 69.886 | 86.065 |
| 432.371 | 76.6031 | 83.921 | 79.7227 |
| 436.099 | 67.3075 | 69.9856 | 69.7061 |
| 439.826 | 66.5262 | 76.7083 | 88.8918 |
| 443.553 | 65.147 | 58.9687 | 106.963 |
| 447.281 | 79.3914 | 71.3061 | 100.793 |
| 451.008 | 77.1146 | 73.3884 | 85.638 |
| 454.735 | 69.6634 | 72.8537 | 90.9144 |
| 458.463 | 57.9766 | 70.9051 | 111.285 |
| 462.19 | 65.3364 | 82.8144 | 90.892 |
| 465.917 | 56.9789 | 75.043 | 69.6482 |
| 469.645 | 59.3972 | 74.5129 | 72.6036 |
| 473.372 | 52.5562 | 83.9386 | 89.5636 |
| 477.099 | 61.8154 | 84.8644 | 71.6301 |
| 480.827 | 41.8364 | 70.0221 | 49.9738 |
| 484.554 | 41.325 | 82.4644 | 62.1974 |
| 488.281 | 61.3863 | 82.8773 | 75.9821 |
| 492.009 | 55.507 | 80.1326 | 77.5788 |
| 495.736 | 31.2799 | 59.6584 | 80.3326 |
| 499.463 | 49.9232 | 63.73 | 86.1719 |
| 503.191 | 42.3303 | 70.889 | 61.1139 |
| 506.918 | 62.6537 | 85.5313 | 45.7992 |
| 510.645 | 65.1388 | 65.2248 | 43.6919 |
| 514.373 | 73.5164 | 52.366 | 72.4713 |
| 518.1 | 71.6249 | 44.2781 | 66.1519 |
| 521.827 | 66.3205 | 75.0343 | 52.1998 |
| 525.555 | 53.0985 | 54.0132 | 40.5971 |
| 529.282 | 71.3179 | 58.7758 | 74.5499 |
| 533.009 | 83.7792 | 61.1176 | 102.44 |
| 536.737 | 76.8155 | 66.7428 | 94.7455 |
| 540.464 | 78.0636 | 69.2275 | 82.0721 |
| 544.191 | 83.4227 | 70.0645 | 81.8694 |
| 547.919 | 77.2644 | 52.0017 | 78.7908 |
| 551.646 | 72.2347 | 57.8901 | 88.7099 |
| 555.373 | 69.8397 | 57.6825 | 115.697 |
| 559.101 | 87.9382 | 66.2875 | 99.5861 |
| 562.828 | 75.6752 | 81.2021 | 75.8371 |
| 566.555 | 64.9104 | 65.3684 | 72.6502 |
| 570.283 | 84.0728 | 64.4105 | 69.5338 |
| 574.01 | 83.2712 | 83.4432 | 82.9025 |
| 577.737 | 76.2205 | 71.1535 | 83.1722 |
| 581.465 | 86.5546 | 74.5694 | 79.8593 |
| 585.192 | 92.003 | 70.3984 | 81.3216 |
| 588.919 | 80.7274 | 74.3184 | 81.1647 |
| 592.647 | 82.4182 | 76.1043 | 81.1953 |
| 596.374 | 90.0653 | 76.7522 | 77.8635 |
| 600.101 | 89.6744 | 78.0112 | 76.5586 |
| 603.829 | 87.3229 | 75.2766 | 76.6036 |
| 607.556 | 84.9108 | 72.3671 | 75.6498 |
| 611.283 | 84.3874 | 70.5199 | 74.395 |
| 615.011 | 88.625 | 71.9455 | 75.507 |
| 618.738 | 90.2637 | 74.0371 | 76.2424 |
| 622.465 | 89.0575 | 73.5773 | 76.907 |
| 626.193 | 89.1143 | 73.31 | 77.5278 |
| 629.92 | 89.6501 | 73.4687 | 76.2954 |
| 633.647 | 89.7945 | 72.6191 | 74.2597 |
| 637.375 | 90.1136 | 71.6202 | 74.299 |
| 641.102 | 90.0366 | 71.5175 | 75.054 |
| 644.829 | 90.5771 | 71.961 | 74.7108 |
| 648.557 | 90.2222 | 72.0958 | 72.5689 |
| 652.284 | 88.8846 | 71.4792 | 66.518 |
| 656.011 | 83.0244 | 70.5769 | 58.864 |
| 659.739 | 74.1283 | 71.1155 | 57.3542 |
| 663.466 | 74.0264 | 73.3596 | 61.2813 |
| 667.193 | 83.9437 | 75.4581 | 63.3717 |
| 670.921 | 90.9967 | 76.6516 | 64.5665 |
| 674.648 | 92.0121 | 77.0582 | 70.0164 |
| 678.375 | 92.1936 | 77.1116 | 74.7462 |
| 682.103 | 92.2124 | 77.2715 | 75.5111 |
| 685.83 | 92.0369 | 77.2041 | 73.5484 |
| 689.558 | 91.7628 | 77.1015 | 70.4699 |
| 693.285 | 91.395 | 77.1984 | 69.432 |
| 697.012 | 90.8778 | 77.1783 | 70.0323 |
| 700.74 | 90.0473 | 76.8765 | 68.8467 |
| 704.467 | 89.0341 | 76.6618 | 65.0998 |
| 708.194 | 87.7006 | 76.3654 | 57.0738 |
| 711.922 | 86.4194 | 75.9781 | 44.2055 |
| 715.649 | 84.6936 | 75.4979 | 34.7333 |
| 719.376 | 82.0682 | 74.8086 | 36.8876 |
| 723.104 | 77.979 | 73.6526 | 45.2396 |
| 726.831 | 71.5114 | 71.6577 | 50.7294 |
| 730.558 | 61.969 | 68.1781 | 49.7309 |
| 734.286 | 51.0173 | 63.4841 | 43.8462 |
| 738.013 | 41.9115 | 59.3461 | 37.0311 |
| 741.74 | 36.6542 | 56.9159 | 32.1637 |
| 745.468 | 34.945 | 55.7189 | 30.1382 |
| 749.195 | 36.9852 | 56.1256 | 31.4675 |
| 752.922 | 42.5595 | 58.9934 | 36.0327 |
| 756.65 | 50.9448 | 64.1381 | 43.2698 |
| 760.377 | 60.6965 | 69.9601 | 51.8803 |
| 764.104 | 70.3479 | 74.657 | 60.6547 |
| 767.832 | 78.2805 | 77.756 | 68.507 |
| 771.559 | 83.9593 | 79.5616 | 74.4434 |
| 775.286 | 87.4892 | 80.4991 | 77.8018 |
| 779.014 | 89.2801 | 80.8398 | 78.9274 |
| 782.741 | 89.8323 | 80.795 | 78.718 |
| 786.468 | 89.8721 | 80.6341 | 78.2155 |
| 790.196 | 89.8976 | 80.4726 | 78.069 |
| 793.923 | 89.9063 | 80.1976 | 78.237 |
| 797.65 | 89.6656 | 79.8341 | 78.9705 |
| 801.378 | 89.6649 | 79.6693 | 80.5209 |
| 805.105 | 90.2463 | 79.9795 | 82.2036 |
| 808.832 | 91.3869 | 80.5618 | 83.253 |
| 812.56 | 92.5152 | 81.3672 | 83.5261 |
| 816.287 | 93.3698 | 82.5245 | 83.1137 |
| 820.014 | 93.6872 | 83.7273 | 82.1552 |
| 823.742 | 92.0684 | 84.5536 | 81.1672 |
| 827.469 | 87.2434 | 85.0719 | 81.119 |
| 831.196 | 86.7527 | 85.4645 | 82.5014 |
| 834.924 | 91.6495 | 85.7076 | 84.4016 |
| 838.651 | 93.7004 | 85.8814 | 85.562 |
| 842.378 | 93.194 | 86.1686 | 85.5772 |
| 846.106 | 92.6025 | 86.7507 | 85.3553 |
| 849.833 | 92.7791 | 87.3663 | 85.7108 |
| 853.56 | 93.2812 | 87.5349 | 86.3445 |
| 857.288 | 93.388 | 86.9095 | 86.6402 |
| 861.015 | 92.7887 | 85.4579 | 86.4649 |
| 864.742 | 91.4112 | 83.5436 | 85.6821 |
| 868.47 | 88.9141 | 81.3299 | 83.9999 |
| 872.197 | 85.3903 | 78.7822 | 81.6008 |
| 875.924 | 82.067 | 76.2029 | 79.4063 |
| 879.652 | 80.2452 | 74.5696 | 78.2745 |
| 883.379 | 80.3405 | 74.8681 | 78.6071 |
| 887.106 | 81.9923 | 76.9012 | 80.0638 |
| 890.834 | 84.442 | 79.7333 | 82.0568 |
| 894.561 | 87.1414 | 82.7101 | 84.1678 |
| 898.288 | 89.768 | 85.3018 | 86.0814 |
| 902.016 | 91.9505 | 87.0645 | 87.4157 |
| 905.743 | 93.325 | 87.9547 | 88.1532 |
| 909.47 | 93.9543 | 88.3989 | 88.4948 |
| 913.198 | 93.9963 | 88.7686 | 88.4823 |
| 916.925 | 93.5582 | 89.0632 | 88.1794 |
| 920.652 | 92.7796 | 89.042 | 87.7912 |
| 924.38 | 92.1769 | 88.8914 | 87.4868 |
| 928.107 | 92.3775 | 89.0664 | 87.3084 |
| 931.835 | 93.1183 | 89.505 | 87.1003 |
| 935.562 | 93.5948 | 89.7768 | 86.9991 |
| 939.289 | 93.8314 | 89.95 | 87.3685 |
| 943.016 | 94.2968 | 90.234 | 88.1794 |
| 946.744 | 94.8045 | 90.5307 | 88.8619 |
| 950.471 | 94.9817 | 90.6208 | 89.1077 |
| 954.198 | 94.9112 | 90.5637 | 89.0841 |
| 957.926 | 94.7828 | 90.4777 | 89.0036 |
| 961.653 | 94.7401 | 90.4362 | 88.9109 |
| 965.38 | 94.6986 | 90.403 | 88.7931 |
| 969.108 | 94.5206 | 90.3113 | 88.6028 |
| 972.835 | 94.2159 | 90.158 | 88.3819 |
| 976.562 | 93.899 | 89.991 | 88.1741 |
| 980.29 | 93.6994 | 89.8386 | 87.9898 |
| 984.017 | 93.5213 | 89.6711 | 87.7947 |
| 987.745 | 93.182 | 89.4168 | 87.5769 |
| 991.472 | 92.7652 | 88.9964 | 87.3579 |
| 995.199 | 92.3016 | 88.2708 | 87.1007 |
| 998.927 | 91.5813 | 87.1914 | 86.5508 |
| 1002.65 | 90.7152 | 86.1836 | 85.7015 |
| 1006.38 | 90.2748 | 85.8513 | 85.2468 |
| 1010.11 | 90.4109 | 86.1824 | 85.431 |
| 1013.84 | 90.6561 | 86.6216 | 85.3843 |
| 1017.56 | 90.3196 | 86.4299 | 83.8373 |
| 1021.29 | 88.8434 | 85.1834 | 80.5943 |
| 1025.02 | 86.5909 | 83.4291 | 79.7601 |
| 1028.75 | 84.5965 | 82.2315 | 81.2352 |
| 1032.47 | 82.7945 | 81.5017 | 80.6546 |
| 1036.2 | 80.429 | 80.3454 | 78.1702 |
| 1039.93 | 78.2568 | 78.0033 | 74.6831 |
| 1043.65 | 77.607 | 74.7201 | 71.7728 |
| 1047.38 | 78.8395 | 72.1491 | 72.0716 |
| 1051.11 | 81.6377 | 72.2217 | 75.6545 |
| 1054.84 | 84.6172 | 75.6229 | 79.5209 |
| 1058.56 | 86.6749 | 80.1616 | 81.373 |
| 1062.29 | 87.6857 | 82.6789 | 81.5976 |
| 1066.02 | 88.3714 | 83.0451 | 82.5674 |
| 1069.75 | 89.6481 | 82.9873 | 84.9636 |
| 1073.47 | 91.283 | 83.7812 | 87.1678 |
| 1077.2 | 92.4258 | 85.2609 | 88.2809 |
| 1080.93 | 92.9138 | 86.2246 | 88.6745 |
| 1084.66 | 92.9773 | 85.9799 | 88.6597 |
| 1088.38 | 92.7196 | 84.7764 | 88.3079 |
| 1092.11 | 92.2198 | 83.2225 | 87.7238 |
| 1095.84 | 91.7838 | 82.3739 | 87.3023 |
| 1099.56 | 91.3991 | 82.9943 | 87.1244 |
| 1103.29 | 90.7261 | 84.565 | 86.8768 |
| 1107.02 | 90.3618 | 86.3079 | 86.8571 |
| 1110.75 | 91.0635 | 88.0525 | 87.4109 |
| 1114.47 | 92.2503 | 89.4216 | 88.0906 |
| 1118.2 | 93.2051 | 90.2908 | 88.6615 |
| 1121.93 | 93.7185 | 90.7424 | 88.9779 |
| 1125.66 | 93.7065 | 90.8146 | 88.982 |
| 1129.38 | 93.4789 | 90.7456 | 88.9938 |
| 1133.11 | 93.3881 | 90.7511 | 89.1414 |
| 1136.84 | 93.1416 | 90.6128 | 89.0537 |
| 1140.57 | 92.2923 | 90.0265 | 88.4415 |

**Figure S3**: Superimposed FTIR spectra of [DOS] and [SaL] based p-anisidinium ISs
